# Deep evolutionary fusion neural network: a new prediction standard for infectious disease incidence rates

**DOI:** 10.1186/s12859-023-05621-5

**Published:** 2024-01-23

**Authors:** Tianhua Yao, Xicheng Chen, Haojia Wang, Chengcheng Gao, Jia Chen, Dali Yi, Zeliang Wei, Ning Yao, Yang Li, Dong Yi, Yazhou Wu

**Affiliations:** 1 Department of Health Statistics, College of Preventive Medicine, Army Medical University, NO.30 Gaotanyan Street, Shapingba District, Chongqing, 400038 China; 2 Department of Health Education, College of Preventive Medicine, Army Medical University, NO.30 Gaotanyan Street, Shapingba District, Chongqing, 400038 China

**Keywords:** Infectious diseases, Time series, Neural network, Neuroevolution, Meta-heuristic algorithm

## Abstract

**Background:**

Previously, many methods have been used to predict the incidence trends of infectious diseases. There are numerous methods for predicting the incidence trends of infectious diseases, and they have exhibited varying degrees of success. However, there are a lack of prediction benchmarks that integrate linear and nonlinear methods and effectively use internet data. The aim of this paper is to develop a prediction model of the incidence rate of infectious diseases that integrates multiple methods and multisource data, realizing ground-breaking research.

**Results:**

The infectious disease dataset is from an official release and includes four national and three regional datasets. The Baidu index platform provides internet data. We choose a single model (seasonal autoregressive integrated moving average (SARIMA), nonlinear autoregressive neural network (NAR), and long short-term memory (LSTM)) and a deep evolutionary fusion neural network (DEFNN). The DEFNN is built using the idea of neural evolution and fusion, and the DEFNN + is built using multisource data. We compare the model accuracy on reference group data and validate the model generalizability on external data. (1) The loss of SA-LSTM in the reference group dataset is 0.4919, which is significantly better than that of other single models. (2) The loss values of SA-LSTM on the national and regional external datasets are 0.9666, 1.2437, 0.2472, 0.7239, 1.4026, and 0.6868. (3) When multisource indices are added to the national dataset, the loss of the DEFNN + increases to 0.4212, 0.8218, 1.0331, and 0.8575.

**Conclusions:**

We propose an SA-LSTM optimization model with good accuracy and generalizability based on the concept of multiple methods and multiple data fusion. DEFNN enriches and supplements infectious disease prediction methodologies, can serve as a new benchmark for future infectious disease predictions and provides a reference for the prediction of the incidence rates of various infectious diseases.

## Background

The incidence of infectious diseases has always been a public health problem worthy of attention and can cause very large social, economic and health burdens [1]. Using data on the numbers of infectious cases to predict the trends of infectious diseases can provide a focus and direction for the actual prevention and control of infectious diseases and can also allow for the evaluation of epidemic prevention effects and long-term outcomes [2]. However, previous research on infectious disease prediction has mainly focused on a single disease and a single region, and there have been few systematic studies on multiple national and regional diseases [3–5]. Therefore, in this paper, the effectiveness and universality of the incidence rates of infectious diseases in nationwide and regional epidemics are studied.

At present, there are many problems, such as poor model accuracy and weak generalization performance, in the field of infectious disease trend prediction [6]. There are many kinds of time series prediction models for infectious diseases, and there is no systematic, unified and standardized research or modelling approach. Previous studies often use time series methods in the prediction of incidence rates of infectious diseases.

Autoregressive integrated moving average (ARIMA) uses the historical values ​​of a univariate time series to predict future values ​​and is suitable for processing stationary data with linear trends. On this basis, the seasonal ARIMA (SARIMA) model was also developed. SARIMA fully considers seasonal information to effectively predict seasonal infectious diseases [7]. However, SARIMA presupposes the basic time series to be linear, so it is not suitable for analysing data containing nonlinear time series [8]. To solve this problem, nonlinear machine learning models represented by artificial neural networks (ANNs) have been gradually proposed and promoted. Nonlinear autoregressive neural networks (NARNNs), ​​hereafter referred to as NARs, approach nonlinear regression through neural networks and can generalize and deal with high-dimensional nonlinear regression estimation [9–12]. In addition, long short-term memory (LSTM), an improved recurrent neural network (RNN), has brought revolutionary changes to various fields. LSTM has powerful feature extraction and representation capabilities, is successful in processing and predicting long and lagging data in time series and can compensate for the defect of vanishing gradients in RNNs [13–15]. Linear statistical models and nonlinear neural network models have their own advantages and disadvantages in time series modelling. The combination of the two models to perform the fusion analysis of infectious disease time series may achieve good results [16].

Previously, data collection for infectious diseases was limited to the diagnosis stage, with issues such as incomplete coverage and poor timeliness. However, the factors influencing the occurrence of infectious diseases are complex, and the traditional monitoring system is ineffective for tracking new infectious diseases. It is now possible to obtain and explore internet data as internet technology advances [17]. The effective use of internet health data may provide high application value, potentially improving the effect of infectious disease early warning research [18]. There are currently some precedents in the use of internet data in the prediction of infectious diseases [19–21]. The findings highlight the importance and relevance of internet data in the prediction of infectious diseases.

Feature selection and hyperparameter optimization are necessary steps for machine learning methods to achieve good accuracy, but traditional algorithms often have problems, such as slow convergence speeds and the tendency to easily fall into local optima [22]. Evolutionary neural networks (ENNs) are neural network models based on evolutionary computing and neural networks [23]. As a result, in this paper, the concept of evolution is employed to improve the efficiency of hyperparameter estimation, and the coronavirus herd immunity optimizer (CHIO) is used to adjust the hyperparameters [24, 25]. CHIO is a metaheuristic algorithm that was proposed in 2020 and inspired by social distance and a population immune strategy. When the proportion of immunized individuals gradually increases and reaches the group immune state, susceptible individuals are better protected. The metaheuristic algorithm based on evolutionary thinking can effectively improve the optimization accuracy while reducing the optimization time compared to those of the traditional grid search method.

We construct a new deep evolutionary fusion neural network (DEFNN), which aims to fully extract the information of time series by using various types of models. We also select six national or regional external datasets to study the generalizability of the DEFNN. Our method has the following innovations. (1) Multiple methods: We develop a new DEFNN prediction model that combines linear and nonlinear methods based on the residual method and execute it using a meta-heuristic algorithm. The combination of evolutionary ideas can account for the benefits of various types of methods and efficiently search for the optimal hyperparameters. (2) Multisource data: We include infectious disease data as well as internet data, modifying the infectious disease data prediction results and improving the model's prediction ability on the disturbed part. (3) Application value: We are the first to apply the DEFNN model to benchmark data and national and regional external test sets. This model has good prediction accuracy and generalizability. The DEFNN and DEFNN + models constructed in this paper enrich and supplement the methodological research content of infectious disease prediction, serving as new benchmarks for future infectious disease prediction and providing a reference for predicting the incidence of various infectious diseases.

## Methods

**Fig. 1:**
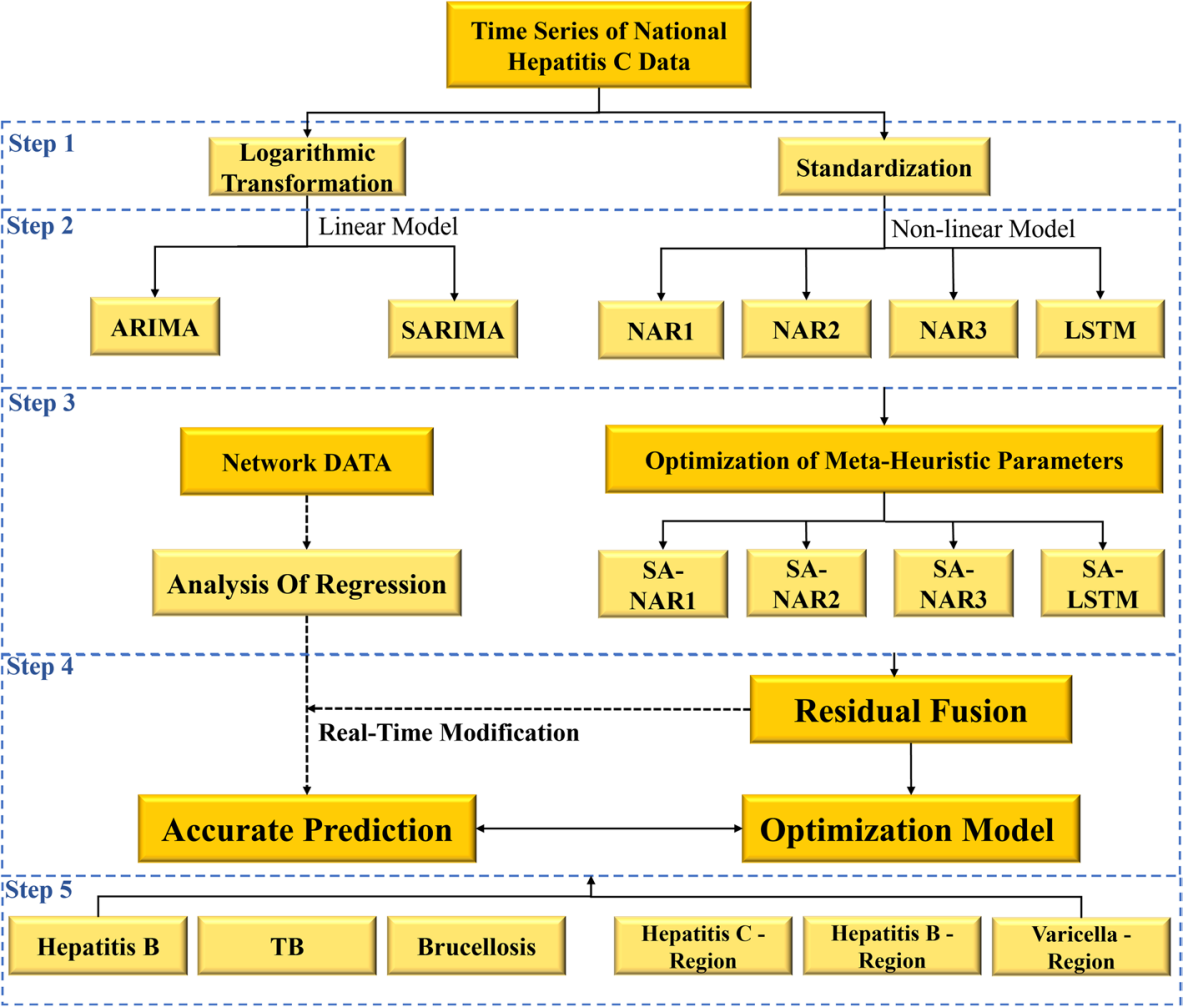
Flowchart of this research. The single models include linear (SARIMA) and nonlinear (NAR and LSTM) models. In NAR, three different algorithms are selected for optimization, including the Levenberg‒Marquardt (LM), scaled convergent gradient (SCG) and Bayesian regulation (BR) algorithms, which are defined as NAR1, NAR2 and NAR3, respectively

Data preprocessing, single model construction, multiple method correction, multisource data correction, generalization performance verification, and other steps are covered in this paper. Figure 1 depicts the technical path of this method.

### Infectious disease data

Seven infectious disease datasets were selected in this paper. The reference group data were the national hepatitis C incidence rate data from the National Health Committee. The national external data included hepatitis B, tuberculosis and brucellosis data, which were obtained from the official website of the National Health Commission. Thr regional external data included hepatitis C-R, hepatitis B-R and varicella-R data, which were obtained from Chongqing Health Commission. The basic information of the datasets is shown in Table 1. The data in this study were reasonably collected, and there were no missing data.

**Table 1:** The basic information of the infectious disease datasets

Application	Dataset	Area	Data source	Time phase (month)	Time phase of training set (month)	Time phase of test set (month)
Reference Group Data	Hepatitis C	National	Official website	2008.1 ~ 2022.4 (172)	2008.1 ~ 2021.4 (160)	2021.5 ~ 2022.4 (12)
National External Data	Hepatitis B	National	Official website	2008.1 ~ 2022.4 (172)	2008.1 ~ 2021.4 (160)	2021.5 ~ 2022.4 (12)
	Tuberculosis	National	Official website	2008.1 ~ 2022.4 (172)	2008.1 ~ 2021.4 (160)	2021.5 ~ 2022.4 (12)
	Brucellosis	National	Official website	2008.1 ~ 2022.4 (172)	2008.1 ~ 2021.4 (160)	2021.5 ~ 2022.4 (12)
Regional External Data	Hepatitis C-R	Chongqing	Database	2008.1 ~ 2022.6 (174)	2008.1 ~ 2021.6 (162)	2021.7 ~ 2022.6 (12)
	Hepatitis B-R	Chongqing	Database	2013.1 ~ 2022.6 (114)	2013.1 ~ 2021.6 (102)	2021.7 ~ 2022.6 (12)
	Varicella-R	Chongqing	Database	2013.1 ~ 2019.12 (84)	2013.1 ~ 2018.12 (72)	2019.1 ~ 2019.12 (12)

R stands for regional dataset, the time span count (month) is in parentheses, the official website refers to the official website of the Chinese Health Commission, and the database refers to the database of the Health Commission

In addition, population data were sourced from China's annual statistical yearbooks, and geographic data were sourced from the National Basic Geographic Information Database.

### Internet data

The internet data are sourced from the Baidu index platform, which mainly focuses on the search frequency and click frequency of key texts. The internet retrieval keywords are shown in Table 2. The Baidu information index is used to measure the attention and popularity of a specific news topic in a Baidu search over a certain period. It is based on big data analysis and search click-through volume, reflecting the popularity and level of attention of a specific news topic during a specific period.

**Table 2:** Search keywords of internet data

Infectious Disease	Keywords
Hepatitis C	Hepatitis C, hepatitis, class B infectious disease, transmission
Hepatitis B	Hepatitis B, hepatitis, class B infectious disease, transmission
Tuberculosis	Tuberculosis, cough, class B infectious disease, transmission
Brucellosis	Brucellosis, infectious disease, class B infectious disease, transmission

The basic information for feature extraction and selection of internet data is shown in Table 3. Feature extraction is performed on each index time series, 22 representative features are selected for each index, and variance selection is performed with a threshold of 1.0. The independent variable contains 4 key texts. After extracting time series features from each key text, 22 of the most critical features are selected [26], and 88 features are obtained. Finally, the numbers of features included in hepatitis C, hepatitis B, tuberculosis, and brucellosis datasets are 16, 18, 21, and 22, respectively. Afterwards, we select a decision tree to fit and correct the prediction error of the selected features for the current month. After extracting features from internet data, we complete the conversion from day to month. After conversion, both the independent and dependent variables are monthly data.

**Table 3:** The basic information of the internet data

Infectious Disease	Number of Key Words	Area	Data Source	Time Phase (month)	Number of Time Series Features	Number of Characteristics	Number of Characteristics After Variance Selection
Hepatitis C	4	National	Baidu & Advisory index	2011.1 ~ 2022.4 (136)	22	88	16
Hepatitis B	4	National	Baidu & Advisory index	2011.1 ~ 2022.4 (136)	22	88	18
Tuberculosis	4	National	Baidu & Advisory index	2011.1 ~ 2022.4 (136)	22	88	21
Brucellosis	4	National	Baidu & Advisory index	2011.1 ~ 2022.4 (136)	22	88	22

The Baidu index and consulting index are derived from the Baidu search engine. The specific extraction process to determine the number of time series features is referred to as Catch22

The data acquisition of infectious diseases involves the following steps. First, keyword cooccurrence analysis, expert consensus, and experience are used to determine the highly relevant keyword information of the infectious disease to be analysed through the database. Second, search engines are used to match text data and obtain keyword text index information. The search engine here specifically refers to the Baidu Search Index website, which is beneficial for representing social hotspots and the real-time living conditions of residents. Then, time series feature extraction is performed on the monthly index information to obtain the time series characteristics of each index for each month. Finally, we extract the 22 most important time series features [26, 27] and incorporate them into the model for analysis. Catch22 is a 22 time series feature provided by the feature library in the Hctsa toolbox. This feature set is one of the small time series feature sets with strong predictive ability. By using the Catch22 feature set, we can effectively reduce the computational complexity and avoid feature redundancy without affecting the predictive performance of the final model.

We divided data preprocessing into two parts to improve the convenience of data processing, accelerate the model's convergence speed, and avoid the impact of different dimensions on the accuracy of the results. For linear models, the logarithmic change method was used, and for nonlinear models, the standardization method was used. Thus, the model can conform to a standard normal distribution after data processing.

Furthermore, the model construction, evaluation index, and calculations were all based on MATLAB 2021b, and the geographic information map was drawn using ArcGIS 10.8.1.

### Fusion of multiple methods

**Fig. 2:**
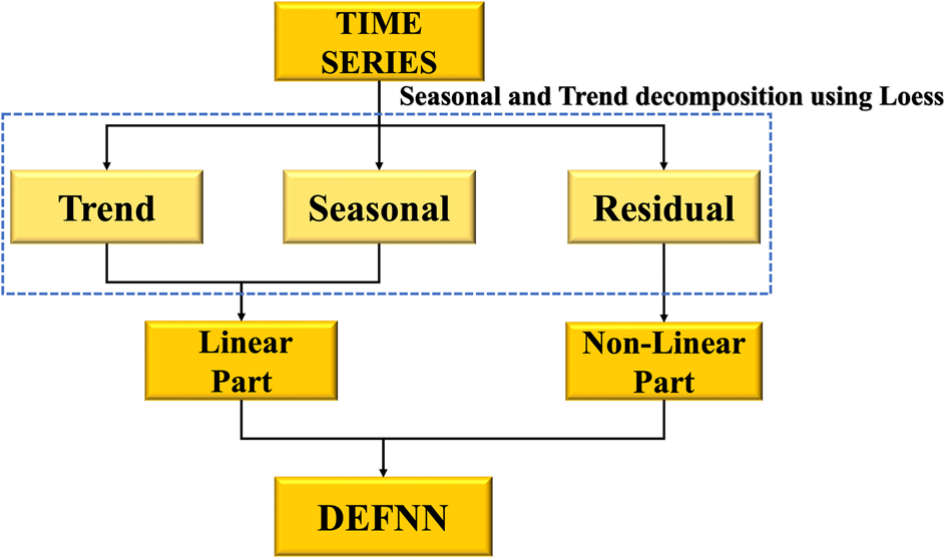
Seasonal-trend decomposition procedure based on loess (STL)

As shown in Fig. 2, the seasonal-trend decomposition procedure based on loess (STL) is a representative time series decomposition algorithm that can divide time series into trend terms, periodic terms, and disturbance terms. The traditional linear time series method is useful for revealing the change rule of the trend and periodic terms, but it does not analyse the disturbance term, which introduces errors into the prediction results. This paper proposes two creative correction methods to better predict the change law of the disturbance term, namely, "multiple methods" and "multisource data."

We examined the nonlinear methods represented by NAR and LSTM, which have a good learning effect for nonlinear characteristics and can effectively evaluate the time series fluctuation state. As a result, we employed nonlinear methods to alter the outcomes of linear time series methods.

Our modelling process incorporates the concepts of "fusion" and "evolution," with the goal of overcoming the limitations of a single method. We used the residual sequence from the linear model as the training set for the nonlinear model, optimized the hyperparameters using deep neural evolution, and then built the DEFNN. The time series prediction model was built using the concept of fusion evolution. The linear and nonlinear model prediction result sequences were fused, and the coronavirus herd immunity optimizer (CHIO) was introduced to optimize the model hyperparameters. Then, the optimization results were obtained [24, 25].

The model was trained using reference group data (hepatitis C dataset). During the training process, CHIO was used to perform hyperparameter optimization, after which the best hyperparameters of various models could be determined. Details can be found in Table 4. After training, the national and regional external datasets were used to validate the optimal model's generalization performance. Finally, we built the deep evolutionary fusion neural network, which included SA-NAR-1, SA-NAR-2, SA-NAR-3, and SA-LSTM, and tested it.

**Table 4:** Various model hyperparameters and their optimal values

Method	Hyperparameters	Values
SARIMA	(p, d, q)	(0,1,1)
	(P, D, Q)	(0,1,1)
NAR1	No. of Neurons	50
	Time Step	12
NAR2	No. of Neurons	30
	Time Step	12
NAR3	No. of Neurons	50
	Time Step	13
LSTM	Layers	3
	No. of Neurons	180
	Learning Rate	0.005
	Batch Size	5
	Epochs	200
	Time Step	12

### Fusion of multisource data

**Fig. 3:**
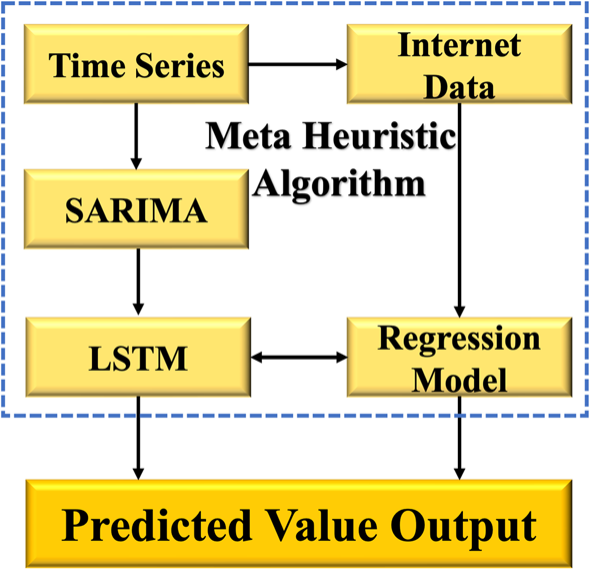
Fusion of multisource data (time series data + internet data)

The traditional incidence rate report has a lag effect, which affects the time series prediction accuracy. Internet data are useful for providing proactive information and overcoming the lag effect associated with traditional data. As a result, we used internet data to modify the results. The residual fusion process produces the prediction results, and the model performance is compared. Simultaneously, network data from specific infectious diseases can be used for the regression prediction of the residual part, resulting in an accurate prediction. Finally, the DEFNN + was built on top of the DEFNN. The distinction is whether internet data were used. Figure 3 depicts the multisource data fusion process.

In this paper, multisource data fusion was implemented on the national dataset to study the improvement effect of network big data correction on the prediction results. Regression analysis was carried out with internet data as the independent variable and the residual after model fitting as the dependent variable. We also carried out an ex-right operation on the internet data, i.e., dividing the Baidu index by the overall index activity of the month, to eliminate the impact caused by the fluctuation of the number of search engine users.

### Evaluation metrics

We used five common evaluation indicators, including the mean square error (MSE), mean absolute error (MAE), root MSE (RMSE), mean absolute percentage error (MAPE) and R-square (*R*^2^) metrics. The first four indicators represent the model fitting error, while *R*^2^ represents the model fitting trend. The smaller the error value is, the better the fitting performance of the model, and the greater the *R*^2^ is, the stronger the ability of the model to predict the trend of actual data. In addition, to better evaluate the accurate proportion of rising or falling trends in the prediction of infectious diseases at each time node, this paper creatively proposes the concept of the accuracy of trend prediction (ATP). $n_{i}$ represents whether the predicted trend is accurate during the _*ith*_ prediction and can be expressed as

1 $$n_{i} = \left\{ {\begin{array}{*{20}c} {1;(\hat{y}_{i} - y_{i - 1} )/(y_{i} - y_{i - 1} ) > 0} \\ {0;(\hat{y}_{i} - y_{i - 1} )/(y_{i} - y_{i - 1} ) < 0} \\ \end{array} } \right.$$

where the last value of $y_{0}$ is the validation set. When $i$ > 0, $y_{i}$ represents the test set sequence, $\hat{y}_{i}$ represents the prediction results of the model on the test set, and $y_{i} \ne y_{i - 1}$. Hence, the ATP can be defined as

2 $$ATP = \frac{{\sum\limits_{i = 1}^{N} {n_{i} } }}{N} \times 100\%$$

where *N* represents the number of test set total time nodes.

To comprehensively utilize the MSE, *R*^2^, and ATP evaluation indicators, we constructed a joint objective function named $loss_{J}$, and its calculation method is defined as

3 $${\text{min}}\;loss_{J} = \frac{{\left[ {MSE + (1 - R^{2} )} \right]}}{ATP}$$

Our objective function ensures that the sought parameters minimize the error (MSE) and maximize the degree of fit (*R*^2^) between the model's predicted data and actual data, thereby comprehensively balancing the accuracy of the built model in predicting data and predicting trends in infectious disease incidence. When using optimization algorithms for hyperparameter optimization, the use of joint objective functions can simultaneously provide direction and step size guidance for the next iteration of the algorithm by the MSE, *R*^2^, and ATP, avoiding single indicator variation errors or premature convergence affecting the final optimal hyperparameter determination. In addition, when constructing the final DEFNN + model, to better perform residual fusion and avoid overfitting, we also used fivefold cross validation to adjust the model parameters.

## Results

### Time and spatial descriptions

**Fig. 4:**
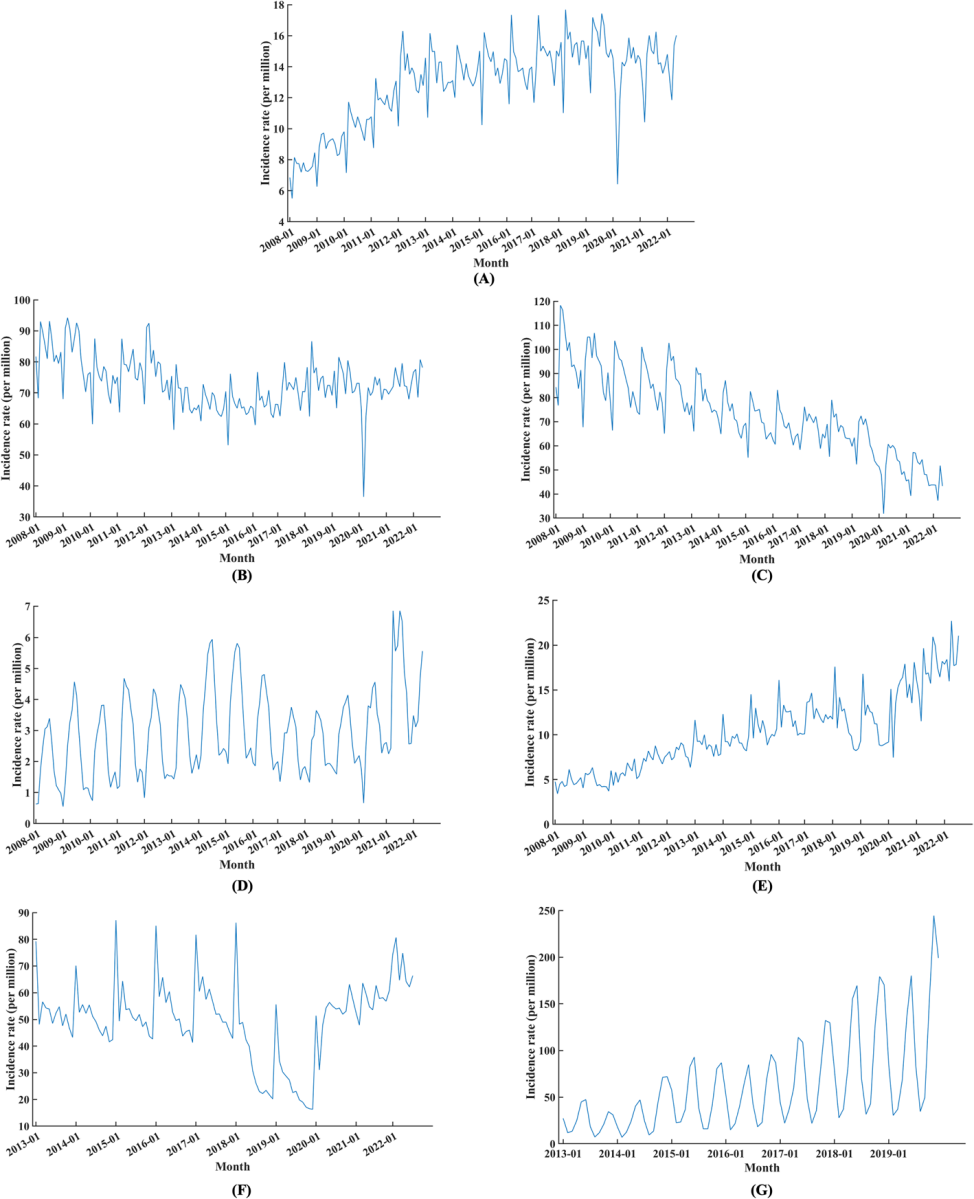
Time trends of the datasets. a: Hepatitis C; b: Hepatitis B; c: Tuberculosis; d: Brucellosis; e: Hepatitis C-R; f: Hepatitis B-R; g: Varicella-R

**Fig. 5:**
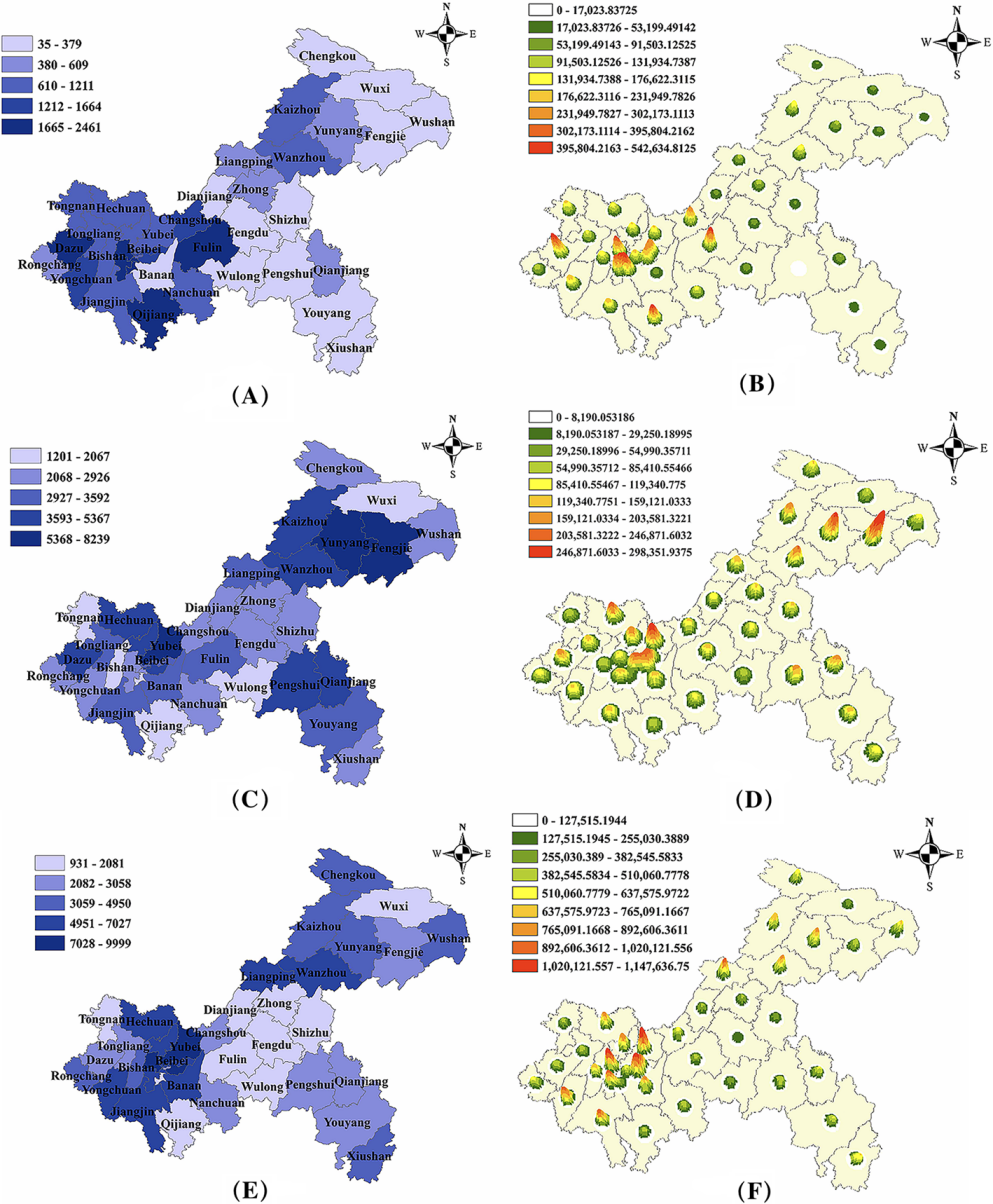
Spatial distributions of the regional external datasets. Our base map is based on the standard map with the review number GS (2019) 3333 downloaded from the Standard Map Service website of the National Bureau of Surveying and Mapping Geographic Information. The base map has not been modified. **a** ~ **b**: Hepatitis C-R, **c** ~ **d**: Hepatitis B-R, **e** ~** f**: Varicella-R; **a**, **c**, **e**: Geographical Plots, **b**, **d**, **f**: Kernel Density Plots

Here, the temporal and spatial epidemic characteristics of the seven datasets are described. Figure 4 shows the time trend diagram, and Fig. 5 shows the spatial distribution diagram. The overall development trend has certain seasonal characteristics.

### Prediction accuracy of the DEFNN

**Fig. 6:**
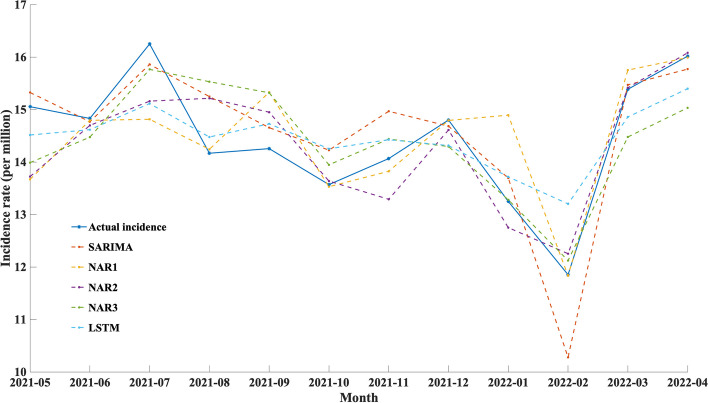
Prediction effect of each single model on the reference group data

**Fig. 7:**
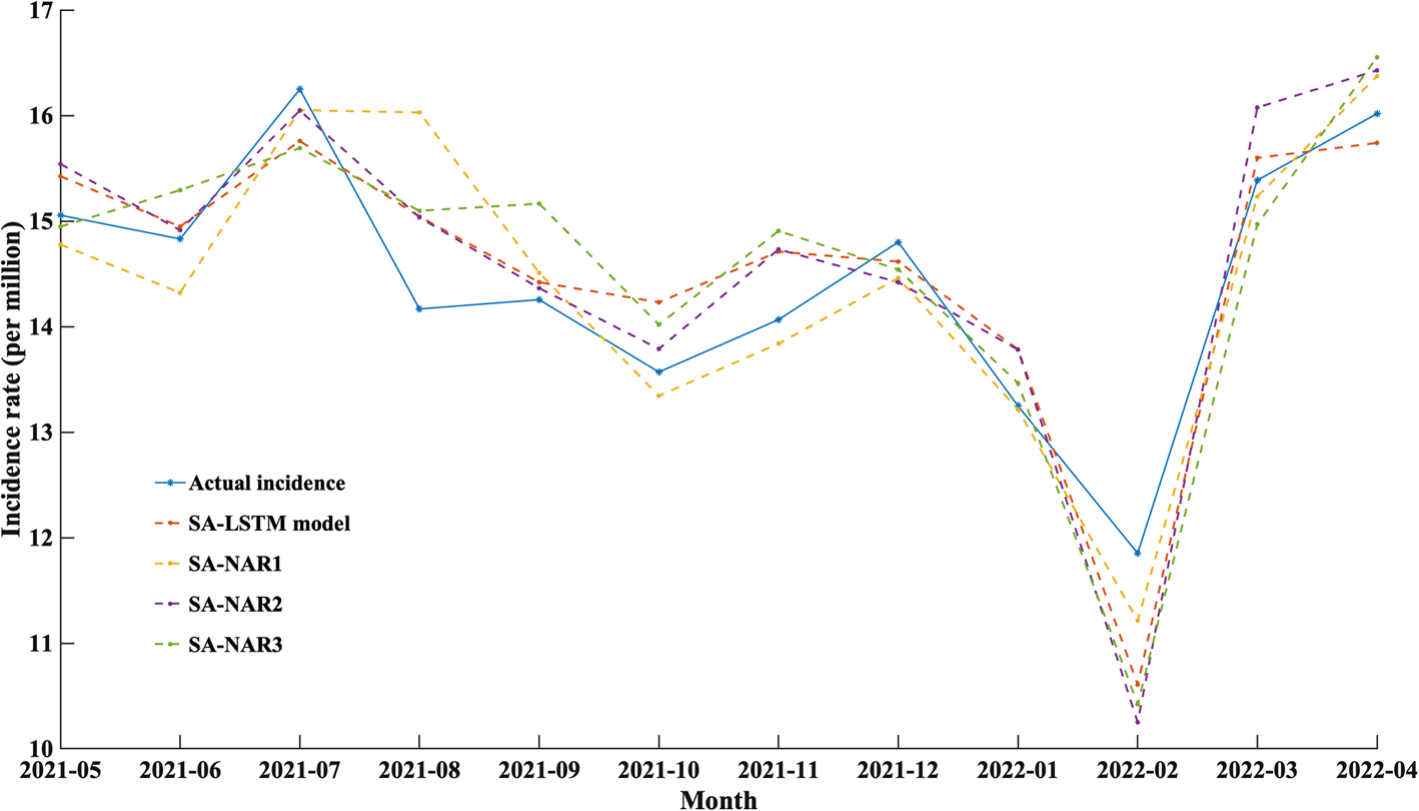
Prediction effect of each fusion model on the reference group data

Five single models and four fusion models were selected for training and testing on the reference group data. The single models involved in the comparison include SARIMA, NAR1, NAR2, NAR3, and LSTM, and the DEFNN models involved in the comparison include SA-LSTM, SA-NAR1, SA-NAR2, and SA-NAR3. The prediction effect of each model on the reference group data is shown in Table 5, Figs. 6, and 7, and only the test set data are shown.

**Table 5:** Prediction effect of the single and fusion models on the reference group data

Model	MSE	MAE	RMSE	MAPE	R^2^	ATP	${\varvec{loss}}_{{\varvec{J}}}$
SARIMA	0.1965	0.5192	0.6807	0.0382	0.6617	0.7500	0.7131
NAR1	0.2362	0.5292	0.8183	0.0363	0.5110	0.7500	1.0069
NAR2	0.1974	0.5265	0.6839	0.0364	0.6585	0.5833	0.9239
NAR3	0.2188	0.6487	0.7581	0.0441	0.5804	0.6667	0.9575
LSTM	0.1952	0.5973	0.6760	0.0422	0.6663	0.7500	0.7053
SA-NAR1	0.1803	0.4229	0.6247	0.0299	0.7151	0.8333	0.5583
SA-NAR2	0.1896	0.5206	0.6568	0.0378	0.6850	0.8333	0.6055
SA-NAR3	0.2000	0.5930	0.6927	0.0425	0.6496	0.7500	0.7339
SA-LSTM	0.1673	0.4816	0.5796	0.0350	0.7574	0.8333	0.4919

SARIMA, NAR1, NAR2, NAR3 and LSTM are single models, while SA-NAR1, SA-NAR2, SA-NAR3 and SA-LSTM are DEFNN models. MSE: mean square error, MAE: mean absolute error, RMSE: root mean square error, R2: R-square, ATP: accuracy of trend prediction

For the single models, the MSE, MAE, RMSE and $loss_{J}$ values of SARIMA are all lower than those of the other models, while the *R*^2^ value is better than those of the other models; hence, it is the best single model. Compared with the single models, the indicators of the DEFNN are improved to varying degrees. On the test set, the MSE, MAE, RMSE and $loss_{J}$ values of the SA-LSTM model are all lower than those of the other models, while the *R*^2^ value is better than those of the other models. The $loss_{J}$ value of SA-LSTM is 0.4919. The SA-LSTM model is conducive to improving the overall prediction ability of the SA-LSTM model and reducing the gap between the predicted and actual values. SA-LSTM is the optimal model for our infectious disease trend prediction task.

### Prediction generalization of the DEFNN

**Fig. 8:**
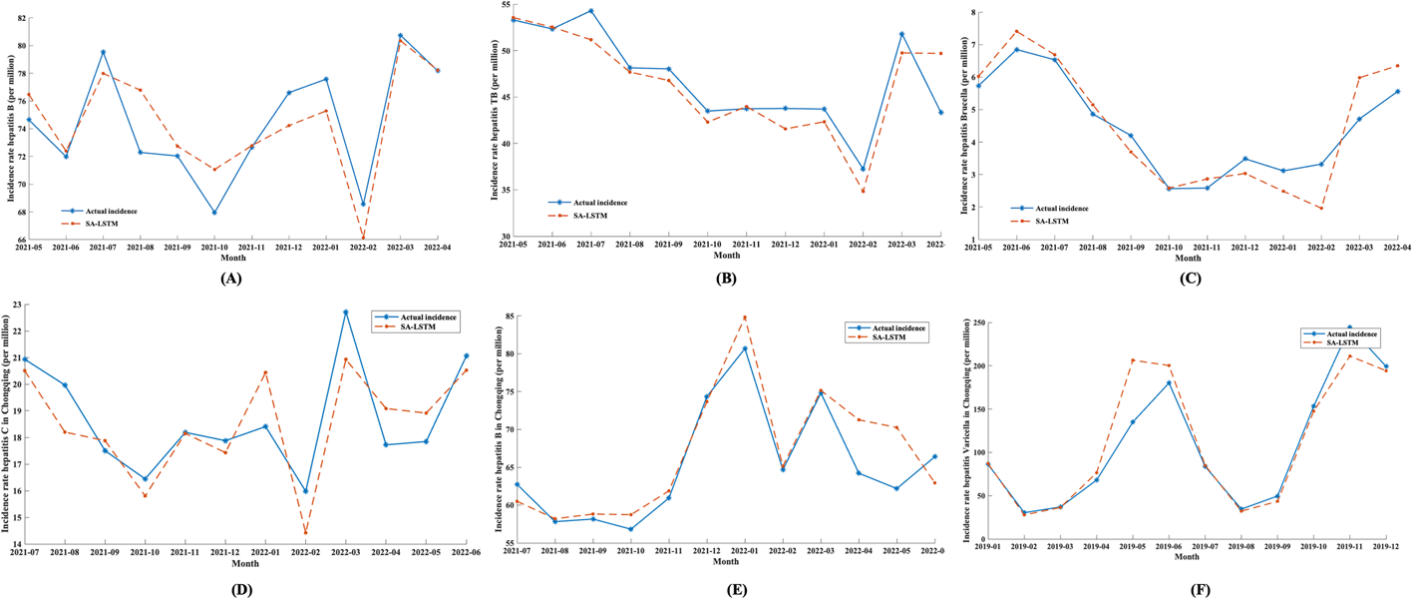
Prediction results of the optimal model on the national external validation data. **a**: Hepatitis B; **b**: Tuberculosis; **c**: Brucellosis; **d**: Hepatitis C-R; **e**: Hepatitis B-R; **f**: Varicella-R

The optimal SA-LSTM model was selected using the reference group data. To analyse the robustness and generalizability of the optimal model on various datasets, three national external validation datasets and three regional external validation datasets were selected for validation. See Table 6 and Fig. 8 for the results. The $loss_{J}$ values of SA-LSTM are 0.9666, 1.2437, 0.2472, 0.7239, 1.4026 and 0.6868.

**Table 6:** Prediction results of the DEFNN on the national external validation data

Data	MSE	MAE	RMSE	MAPE	R^2^	ATP	${\varvec{loss}}_{{\varvec{J}}}$
Hepatitis B	0.6074	1.6436	2.1041	0.0225	0.7213	0.9167	0.9666
Tuberculosis	0.6982	1.7561	2.4185	0.0389	0.7654	0.7500	1.2437
Brucellosis	0.1955	0.5487	0.6774	0.1333	0.7734	0.9167	0.2472
Hepatitis C-R	0.3442	1.0028	1.1925	0.0537	0.6203	1.0000	0.7239
Hepatitis B-R	1.0382	2.5307	3.5964	0.0389	0.7522	0.9167	1.4026
Varicella-R	0.6868	1.3131	2.3792	0.8818	0.8333	0.9660	0.6868

Hepatitis B, tuberculosis and brucellosis data are national external data, while Hepatitis C-R, Hepatitis B-R and Varicella-R data are regional external data. MSE: mean square error, MAE: mean absolute error, RMSE: root mean square error, R^2^: R-square, ATP: accuracy of trend prediction

### Prediction accuracy of the DEFNN + 

**Fig. 9:**
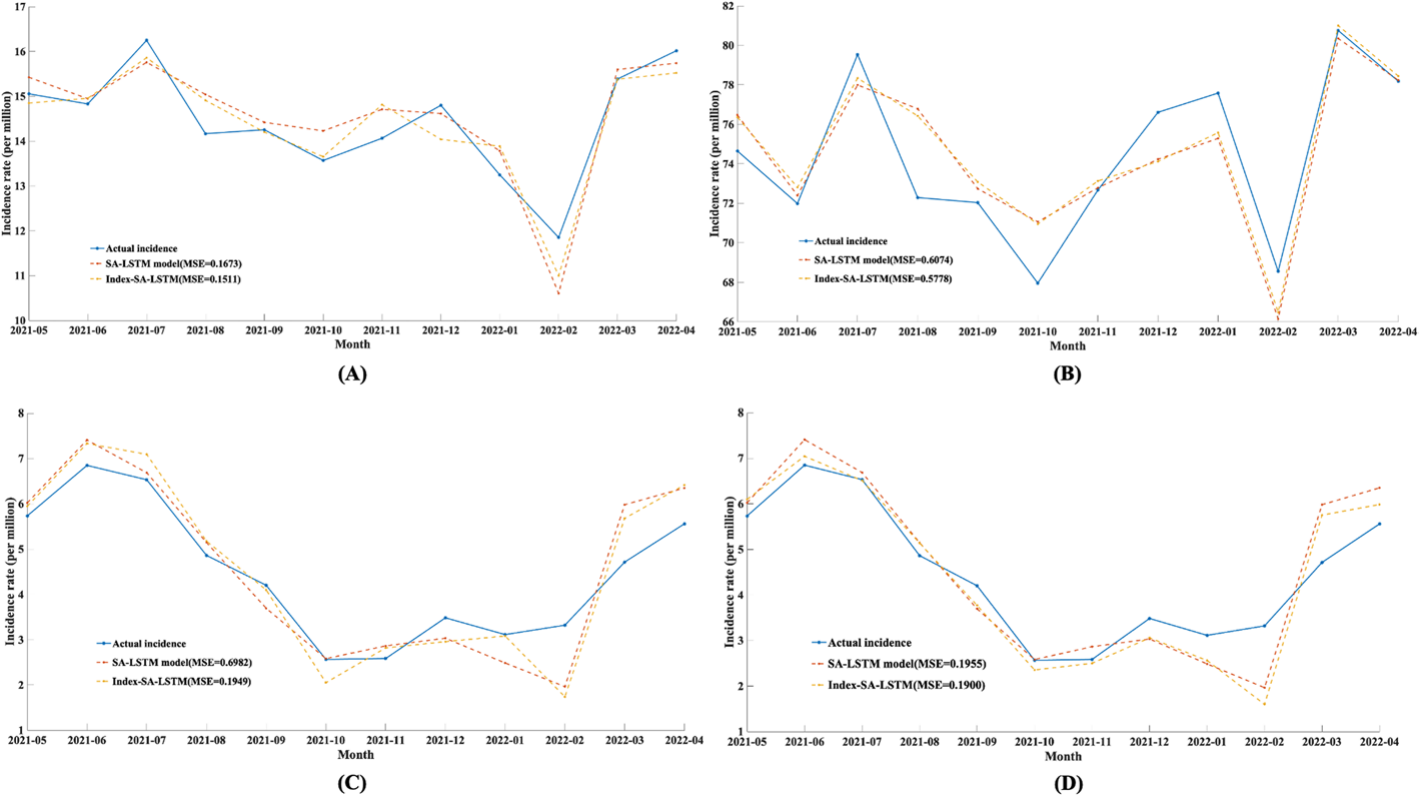
Model effect of the DEFNN + on each dataset. A: Hepatitis B; **b**: Tuberculosis; **c**: Brucellosis; **d**: Hepatitis C-R

We also introduced additional internet data on the basis of SA-LSTM. Table 7 and Fig. 9 display the final prediction results. The results show that using the Baidu index to correct the results and obtain more accurate prediction results is effective. The $loss_{J}$ of the DEFNN + for hepatitis C, hepatitis B, tuberculosis, and brucellosis are 0.4212, 0.8218, 1.0331, and 0.8575, respectively, on the four national datasets.

**Table 7:** Model effect of the DEFNN + on each dataset

Data	MSE	MAE	RMSE	MAPE	R^2^	ATP	${\varvec{loss}}_{{\varvec{J}}}$
Hepatitis C	0.1511	0.4249	0.5233	0.0304	0.8001	0.8333	0.4212
Hepatitis B	0.5714	1.6194	1.9794	0.0222	0.7533	0.9167	0.8218
Tuberculosis	0.1949	0.5321	0.6752	0.1308	0.7748	0.7500	1.0331
Brucellosis	0.1900	0.4790	0.6581	0.1238	0.7861	0.9167	0.8575

### Comparison with previous studies

We compared this method with the most advanced methods in the previous literature to better illustrate the performance of this model. The results show that the prediction performance of this method is better than that of the previous advanced methods. See Table 8 for details.

**Table 8:** Comparison with previous studies

Author	Year	Dataset	MSE	MAE	RMSE	MAPE
Zhang [28]	2014	Hepatitis B	–	–	–	0.1097
Azeez [29]	2016	Tuberculosis	–	–	0.7386	1.1039
Zheng [30]	2020	Hepatitis B	–	–	0.7000	0.8900
Wang [31]	2020	Tuberculosis	–	0.8030	0.9790	0.0765
Guo [32]	2021	Tuberculosis	3.2460	–	–	0.0208
Li [33]	2021	Tuberculosis	–	–	1.1330	0.0796
SA-LSTM	2023	Hepatitis C	0.1673	0.4816	0.5796	0.0350

## Discussion

In the analysis and prediction of the incidence rates of infectious diseases, we compare five single models and propose a fusion evolutionary idea based on the results. We build and select the optimal fusion model and use the national external data and regional external data to verify the optimal model.

SARIMA is a typical representative of linear prediction models. NAR is a representative model of machine learning and has good performance in classification and regression. LSTM is a deep learning model suitable for nonlinear regression [34]. Our DEFNN model effectively fuses the linear part and the nonlinear part through a residual method. When there is a nonlinear part in the prediction data, it can be explained by the neural network, so the effect of the DEFNN is obviously better than that of a single model. In addition, we introduce a function to comprehensively weigh the accuracy of the model in the prediction of infectious diseases, named $loss_{J}$, and then make a more comprehensive evaluation of the performance of the model.

Our results show that SA-LSTM not only has good prediction accuracy but also has good robustness and generalizability in external verification. The reason for these results is that the SARIMA model cannot capture the nonlinear part of time series data, and the evaluation effect of the NAR and LSTM models on the linear part is also limited [35]. The DEFNN can not only make effective use of the seasonal prediction advantages of SARIMA but also explain the nonlinear trend in the dataset [36]. In addition, the evolution process based on a metaheuristic algorithm is conducive to improving the accuracy of the neural network model hyperparameter search [37–39]. The verification results of the external dataset in this paper show that SA-LSTM is suitable not only for the national dataset of a variety of infectious diseases but also for the regional dataset of a variety of infectious diseases.

Internet retrieval data, as represented by the Google influenza index, have gradually been applied to the prediction of infectious diseases [40–42]. Internet search data can effectively reflect population internet behaviour and reasonably measure the population’s spontaneous and proactive search behaviour prior to the onset of infectious diseases. As a result, we introduce the research concept of multisource data fusion and then construct the DEFNN + on the basis of the DEFNN. The DEFNN proposed in this paper is applicable to a wide range of seasonal infectious diseases, has high prediction accuracy, and can be used as a new benchmark in the field of infectious disease trend prediction. In the future, the benchmark that we have created can be used to predict the trend of infectious diseases in more types and regions, providing a better foundation for infectious disease prevention, control, and evaluation.

## Conclusion and future work

In summary, the DEFNN is constructed by using the idea of neuroevolution and fusion. The optimal SA-LSTM model is superior to other models in reference group data prediction and has a good generalization performance on the external test set. The DEFNN + can significantly improve the prediction performance after incorporating internet data. The DEFNN model proposed in this paper has good prediction performance, can provide strong future predictions, and has a strong guiding role in the prevention and control of infectious diseases. It is suggested that this model be extended to predict a variety of seasonal infectious diseases to guide their prevention.

Although the design of this study is reasonable and strictly implemented, there is still some room for improvement. We look forward to the following future research directions: (1) In the future, multidimensional and wide-ranging analyses will be carried out on the characteristics of spatial distributions, population distributions and pathogenic factors to better implement early warnings. (2) In the future, we need to continue to explore the optimization method of model hyperparameters and improve the model effect. (3) The DEFNN + results indicate that the addition of the Baidu index can effectively correct the results and obtain more accurate prediction results, but the effect on the overall trend analysis is limited. As a result, improving the utilization of internet data in the future is required to obtain more accurate prediction results.

## Data Availability

The data of this study are available at the National Health Committee (http://www.nhc.gov.cn/) and Chongqing Health Commission (http://wsjkw.cq.gov.cn). Taking January 2018 as an example, the national infectious disease data source can be found in http://www.nhc.gov.cn/jkj/s6873/201802/4a469b9e02a642d08cc33e855fe134a5.shtml and the Chongqing infectious disease data source can be found in http://wsjkw.cq.gov.cn/zwgk_242/wsjklymsxx/ylws_266434/jbfk_266438/yqxx/202112/t20211209_10112244.html. The datasets analysed during the current study are available from the corresponding author upon reasonable request. The code of this study is available at https://github.com/yaotianhua0924/FENN.

## References

[1] Gitto S, Cursaro C, Bartoli A, Margotti M, Andreone P (2021). Hepatitis C: clinical management and debated issues. Minerva Med.

[2] Guo Y, Feng Y, Qu F, Zhang L, Yan B, Lv J (2020). Prediction of hepatitis E using machine learning models. PLoS ONE.

[3] Ioannou GN, Tang W, Beste LA, Tincopa MA, Su GL, Van T (2020). Assessment of a deep learning model to predict hepatocellular carcinoma in patients with hepatitis C cirrhosis. JAMA Netw Open.

[4] Xu B, Li J, Wang M (2020). Epidemiological and time series analysis on the incidence and death of AIDS and HIV in China. BMC Public Health.

[5] De Brito RJVC, Da Silva LF, Santos MB, De Moura PMMF, De Souza CDF, Do Carmo RF (2022). A time series analysis of detection and mortality of hepatitis C in Brazil, 2008–2018. BMC Infect Dis.

[6] Gupta R, Srivastava D, Sahu M, Tiwari S, Ambasta RK, Kumar P (2021). Artificial intelligence to deep learning: machine intelligence approach for drug discovery. Mol Divers.

[7] Patil S, Pandya S (2021). Forecasting dengue hotspots associated with variation in meteorological parameters using regression and time series models. Front Public Health.

[8] Shahvaroughi Farahani M, Razavi Hajiagha SH (2021). Forecasting stock price using integrated artificial neural network and metaheuristic algorithms compared to time series models. Soft Comput.

[9] Jiang J, Wang H, Xie J, Guo X, Guan Y, Yu Q (2020). Medical knowledge embedding based on recursive neural network for multi-disease diagnosis. Artif Intell Med.

[10] Eikenberry SE, Marmarelis VZ (2013). A nonlinear autoregressive Volterra model of the Hodgkin-Huxley equations. J Comput Neurosci.

[11] Chen S, Yao S (2022). Evaluation and dynamic prediction of ecological security from the perspective of sustainable development: a case study of Shaanxi Province. China Environ Sci Pollut Res.

[12] Lin Y-H (2020). A parallel evolutionary computing-embodied artificial neural network applied to non-intrusive load monitoring for demand-side management in a smart home: towards deep learning. Sensors.

[13] Zhou L, Yu L, Wang Y, Lu Z, Tian L, Tan L (2014). A hybrid model for predicting the prevalence of schistosomiasis in humans of Qianjiang City. China PLoS ONE.

[14] Yu Y, Si X, Hu C, Zhang J (2019). A review of recurrent neural networks: LSTM cells and network architectures. Neural Comput.

[15] Hall EW, Bradley H (2020). Gaps in descriptive epidemiology and hepatitis C virus modeling research. JAMA Netw Open.

[16] Al-Betar MA, Alyasseri ZAA, Awadallah MA, Abu DI (2021). Coronavirus herd immunity optimizer (CHIO). Neural Comput & Applic.

[17] Li C, Chen LJ, Chen X, Zhang M, Pang CP, Chen H (2020). Retrospective analysis of the possibility of predicting the COVID-19 outbreak from Internet searches and social media data China 2020. Eurosurveillance.

[18] Marcelin JR, Cortés-Penfield N, Del Rio C, Desai A, Echenique I, Granwehr B (2021). How the field of infectious diseases can leverage digital strategy and social media use during a pandemic. Open Forum Infect Dis.

[19] Lampos V, Majumder MS, Yom-Tov E, Edelstein M, Moura S, Hamada Y (2021). Tracking COVID-19 using online search. npj Digit Med..

[20] Wang M-Y, Tang N (2021). The correlation between Google trends and salmonellosis. BMC Public Health.

[21] Samaras L, Sicilia M-A, García-Barriocanal E (2021). Predicting epidemics using search engine data: a comparative study on measles in the largest countries of Europe. BMC Public Health.

[22] Zhang R, Gao C, Chen X, Li F, Yi D, Wu Y (2023). Genetic algorithm optimised Hadamard product method for inconsistency judgement matrix adjustment in AHP and automatic analysis system development. Expert Syst Appl.

[23] Hasson U, Nastase SA, Goldstein A (2020). Direct Fit to Nature: An Evolutionary Perspective on Biological and Artificial Neural Networks. Neuron.

[24] Gao C, Zhang R, Chen X, Yao T, Song Q, Ye W (2022). Integrating Internet multisource big data to predict the occurrence and development of COVID-19 cryptic transmission. npj Digit Med..

[25] Martínez-Álvarez F, Asencio-Cortés G, Torres JF, Gutiérrez-Avilés D, Melgar-García L, Pérez-Chacón R (2020). Coronavirus optimization algorithm: a bioinspired metaheuristic based on the COVID-19 propagation model. Big Data.

[26] Lubba CH, Sethi SS, Knaute P, Schultz SR, Fulcher BD, Jones NS (2019). catch22: CAnonical time-series characteristics: selected through highly comparative time-series analysis. Data Min Knowl Disc.

[27] Fulcher BD, Jones NS (2017). hctsa : a computational framework for automated time-series phenotyping using massive feature extraction. Cell Syst.

[28] Zhang L, Zheng Y, Wang K, Zhang X, Zheng Y (2014). An optimized Nash nonlinear grey Bernoulli model based on particle swarm optimization and its application in prediction for the incidence of Hepatitis B in Xinjiang. China Computers in Biology and Medicine.

[29] Azeez A, Obaromi D, Odeyemi A, Ndege J, Muntabayi R (2016). Seasonality and trend forecasting of tuberculosis prevalence data in Eastern Cape, South Africa. Using a Hybrid Model IJERPH.

[30] Zheng Y, Zhang L, Zhu X, Guo G (2020). A comparative study of two methods to predict the incidence of hepatitis B in Guangxi. China PLoS ONE.

[31] Wang Y, Xu C, Li Y, Wu W, Gui L, Ren J (2020). An advanced data-driven hybrid model of SARIMA-NNNAR for tuberculosis incidence time series forecasting in Qinghai Province. China IDR.

[32] Guo X, Shen H, Liu S, Xie N, Yang Y, Jin J (2021). Predicting the trend of infectious diseases using grey self-memory system model: a case study of the incidence of tuberculosis. Public Health.

[33] Li J, Li Y, Ye M, Yao S, Yu C, Wang L (2021). Forecasting the tuberculosis incidence using a novel ensemble empirical mode decomposition-based data-driven hybrid model in Tibet. China IDR.

[34] Wang KW, Deng C, Li JP, Zhang YY, Li XY, Wu MC (2017). Hybrid methodology for tuberculosis incidence time-series forecasting based on ARIMA and a NAR neural network. Epidemiol Infect.

[35] Kırbaş İ, Sözen A, Tuncer AD, Kazancıoğlu FŞ (2020). Comparative analysis and forecasting of COVID-19 cases in various European countries with ARIMA, NARNN and LSTM approaches. Chaos, Solitons Fractals.

[36] Shahid F, Zameer A, Muneeb M (2020). Predictions for COVID-19 with deep learning models of LSTM, GRU and Bi-LSTM. Chaos, Solitons Fractals.

[37] Jiao Y, Gong C, Wang S, Duan Y, Zhang Y (2022). The influence of air pollution on pulmonary disease incidence analyzed based on grey correlation analysis. Contrast Media Mol Imaging.

[38] Song C, Yao L, Hua C, Ni Q (2021). A water quality prediction model based on variational mode decomposition and the least squares support vector machine optimized by the sparrow search algorithm (VMD-SSA-LSSVM) of the Yangtze River. China Environ Monit Assess.

[39] Huang Z, Li H, Huang B (2021). Regional distribution of non-human H7N9 avian influenza virus detections in China and construction of a predictive model. J Veterinary Res.

[40] Kim J, Han J, Chun BC (2022). Trends of internet search volumes for major depressive disorder symptoms during the COVID-19 pandemic in Korea: an interrupted time-series analysis. J Korean Med Sci.

[41] Aiello AE, Renson A, Zivich PN (2020). Social media– and internet-based disease surveillance for public health. Annu Rev Public Health.

[42] Jang B, Kim Y, Il Kim G, Wook KJ (2022). Deep similarity analysis and forecasting of actual outbreak of major infectious diseases using Internet-Sourced data. J Biomed Inform.

